# (*E*)-2-(Benzyl­imino­meth­yl)-4,6-dibromo­phenol

**DOI:** 10.1107/S1600536808019247

**Published:** 2008-07-05

**Authors:** Wei Jiang

**Affiliations:** a College of Chemistry and Life Sciences, Maoming University, Guandu Second Road 139, Maoming 525000, People’s Republic of China

## Abstract

The title compound, C_14_H_11_Br_2_NO, was prepared by the condensation of benzyl­amine and 3,5-dibromo-2-hydroxy­benzaldehyde. The crystal structure is stabilized by aromatic π–π stacking inter­actions between the phenol rings of neighbouring mol­ecules [centroid–centroid distance = 3.530 (5) Å]. In addition, the stacked mol­ecules exhibit inter­molecular C—H⋯π and intra­molecular O—H⋯N inter­actions.

## Related literature

For details of the photochromism and thermochromism of Schiff base compounds, see: Cohen *et al.* (1964 ▶).
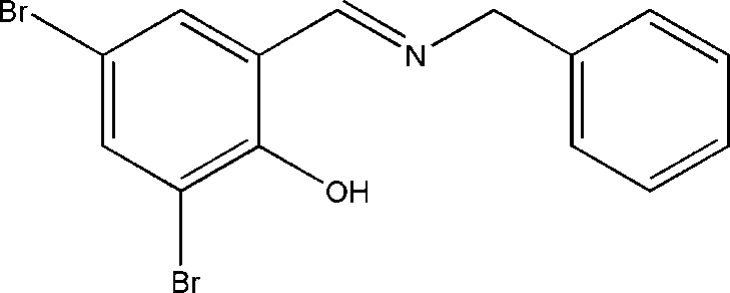


## Experimental

### 

#### Crystal data

C_14_H_11_Br_2_NO
*M* *_r_* = 369.07
Monoclinic, 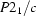

*a* = 12.086 (2) Å
*b* = 8.326 (1) Å
*c* = 13.576 (2) Å
β = 93.126 (2)°
*V* = 1364.1 (3) Å^3^
*Z* = 4
Mo *K*α radiation
μ = 5.93 mm^−1^
*T* = 296 (2) K
0.34 × 0.30 × 0.25 mm

#### Data collection

Bruker APEXII area-detector diffractometer
Absorption correction: multi-scan (*SADABS*; Sheldrick, 2000 ▶) *T*_min_ = 0.156, *T*_max_ = 0.231
11566 measured reflections
3156 independent reflections
2339 reflections with *I* > 2σ(*I*)
*R*_int_ = 0.030

#### Refinement

*R*[*F* ^2^ > 2σ(*F* ^2^)] = 0.030
*wR*(*F* ^2^) = 0.073
*S* = 1.01
3156 reflections
164 parameters
H-atom parameters constrained
Δρ_max_ = 0.45 e Å^−3^
Δρ_min_ = −0.55 e Å^−3^

### 

Data collection: *APEX2* (Bruker, 2004 ▶); cell refinement: *SAINT* (Bruker, 2004 ▶); data reduction: *SAINT*; program(s) used to solve structure: *SHELXS97* (Sheldrick, 2008 ▶); program(s) used to refine structure: *SHELXL97* (Sheldrick, 2008 ▶); molecular graphics: *SHELXTL* (Sheldrick, 2008 ▶); software used to prepare material for publication: *SHELXTL*.

## Supplementary Material

Crystal structure: contains datablocks global, I. DOI: 10.1107/S1600536808019247/lx2061sup1.cif

Structure factors: contains datablocks I. DOI: 10.1107/S1600536808019247/lx2061Isup2.hkl

Additional supplementary materials:  crystallographic information; 3D view; checkCIF report

## Figures and Tables

**Table 1:** Hydrogen-bond geometry (Å, °)

*D*—H⋯*A*	*D*—H	H⋯*A*	*D*⋯*A*	*D*—H⋯*A*
C7—H7*A*⋯*Cg*^i^	0.97	2.88	3.526 (3)	125
O—H1*O*⋯N	0.82	1.88	2.601 (3)	147

Symmetry code: (i) 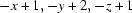. *Cg* is the centroid of the C9–C14 ring.

**Fig. 1.**
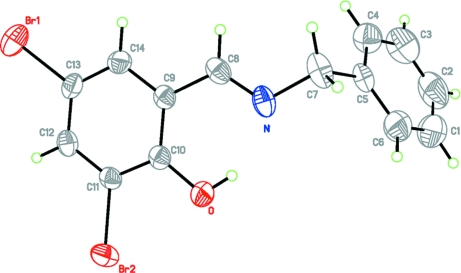
The structure of (I), showing displacement ellipsoids drawn at the 30% probability level.

**Fig. 2.**
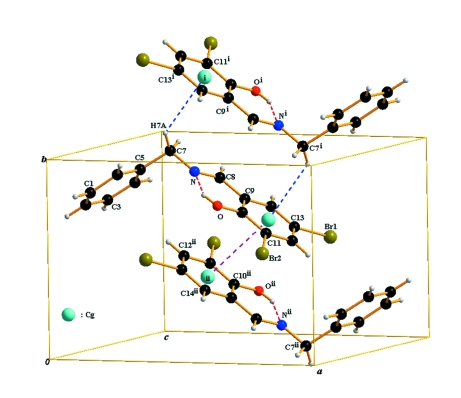
π···π, C—H···π and N—H···O interactions (dotted lines) in the title compound. Cg denotes the ring centroids. [Symmetry code: (i) -x+1, -y+2, -z+1; (ii) -x+1, -y+1, -z+1.]

## supplementary crystallographic information

### Comment

Compounds presenting photochromism, a reversible color change brought about in at least one direction, by the action of electromagnetic radiation, attract considerable attention from various fields of chemistry, physics and material science as potential candidates for practical applications. Since long time,the Schiff bases of salicylaldehyde with aromatic amines (anils or N-salicylideneaniline derivatives) are recognized as such compounds, which undergo enol-keto tautomerism and present common features in their structures and reaction mechanisms.The schiff base compounds show photochromism and thermochromism in the solid state by proton transfer from the hydroxyl O atom to the imine N atom (Cohen *et al.*, 1964). The tautomerism involves proton transfer from the hydroxylic oxygen to the imino nitrogen atom that occurs intramolecularly *via* a six-membered ring, with the keto species showing bathochromically shifted spectra. As our continuing studies on the relation between the Schiff base geometry in the crystalline state and photochromism and/or thermochromism, here we report the crystal structure of the title compound, (E)-benzyliminomethyl-4,6-dibromophenol (Fig. 1).

The molecular structure is a typical salicylaldehyde schiff derivative with normal geometric parameters. The molecular packing (Fig. 2) is stabilized by π—π interactions between the phenol rings of neighbouring molecules. The Cg···Cg^ii^ distance is 3.530 (5) Å (Cg1 is the centroid of the C9-C14 ring, symmetry code as in Fig. 2). The crystal packing (Fig. 2) is further stabilized by the C—H···π interactions between a methylene H atom of the benzyl group and the phenol ring, i.e. with a C7–H7A···Cg^i^ separation of 2.88 Å (Fig. 2 and Table 1; symmetry code as in Fig. 2). Additionally, intramolecular O—H···N interactions in the structure were observed (Fig. 2 and Table 1; symmetry code as in Fig. 2).

### Experimental

Benzylamine (0.02 mol, 2.14 g) and 3,5-dibromo-2-hydroxybenzaldehyde(0.02 mol, 5.498 g) were dissolved in ethanol and the solution was refluxed for 3 h. After evaporation, a crude product was recrystallized twice from ethanol to give a pure yellow product. Yield: 82.5%. Calcd. for C_14_H_11_Br_2_NO: C, 45.56; H, 3.00; N, 3.80; Found: C, 45.21; H, 2.858; N, 3.67%.

### Refinement

All H atoms were located from difference Fourier syntheses, H atoms from the C—H groups and O—H group were placed in geometrically idealized positions and constrained to ride on their parent atoms (C—H = 0.93 %A, 0.96 %A, 0.97 %A; O—H = 0.82 Å) and *U*_iso_(H) values equal to 1.2 *U*_eq_(C) or 1.5Ueq(O).

### Crystal data

**Table:**

C_14_H_11_Br_2_NO	*F*_000_ = 720
*M**_r_* = 369.07	*D*_x_ = 1.797 Mg m^−^^3^
Monoclinic, *P*2_1_/*c*	Mo *K*α radiation λ = 0.71073 Å
Hall symbol: -P 2ybc	Cell parameters from 1859 reflections
*a* = 12.086 (2) Å	θ = 1.0–27.6º
*b* = 8.326 (1) Å	µ = 5.93 mm^−^^1^
*c* = 13.576 (2) Å	*T* = 296 (2) K
β = 93.126 (2)º	Block, yellow
*V* = 1364.1 (3) Å^3^	0.34 × 0.30 × 0.25 mm
*Z* = 4	

### Data collection

**Table:**

Bruker APEXII area-detector diffractometer	3156 independent reflections
Radiation source: fine-focus sealed tube	2339 reflections with *I* > 2σ(*I*)
Monochromator: graphite	*R*_int_ = 0.030
Detector resolution: 10.00 pixels mm^-1^	θ_max_ = 27.6º
*T* = 296(2) K	θ_min_ = 1.7º
φ and ω scans	*h* = −15→15
Absorption correction: multi-scan(SADABS; Sheldrick, 2000)	*k* = −10→10
*T*_min_ = 0.156, *T*_max_ = 0.231	*l* = −17→17
11566 measured reflections	

### Refinement

**Table:**

Refinement on *F*^2^	Hydrogen site location: inferred from neighbouring sites
Least-squares matrix: full	H-atom parameters constrained
*R*[*F*^2^ > 2σ(*F*^2^)] = 0.030	*w* = 1/[σ^2^(*F*_o_^2^) + (0.0301*P*)^2^ + 0.6854*P*] where *P* = (*F*_o_^2^ + 2*F*_c_^2^)/3
*wR*(*F*^2^) = 0.073	(Δ/σ)_max_ = 0.001
*S* = 1.01	Δρ_max_ = 0.45 e Å^−^^3^
3156 reflections	Δρ_min_ = −0.55 e Å^−^^3^
164 parameters	Extinction correction: SHELXL97 (Sheldrick, 2008), Fc^*^=kFc[1+0.001xFc^2^λ^3^/sin(2θ)]^-1/4^
Primary atom site location: structure-invariant direct methods	Extinction coefficient: 0.0030 (4)
Secondary atom site location: difference Fourier map	

### Special details

**Table:**

Geometry. All esds (except the esd in the dihedral angle between two l.s. planes) are estimated using the full covariance matrix. The cell esds are taken into account individually in the estimation of esds in distances, angles and torsion angles; correlations between esds in cell parameters are only used when they are defined by crystal symmetry. An approximate (isotropic) treatment of cell esds is used for estimating esds involving l.s. planes.
Refinement. Refinement of F^2^ against ALL reflections. The weighted R-factor wR and goodness of fit S are based on F^2^, conventional R-factors R are based on F, with F set to zero for negative F^2^. The threshold expression of F^2^ > 2sigma(F^2^) is used only for calculating R-factors(gt) etc. and is not relevant to the choice of reflections for refinement. R-factors based on F^2^ are statistically about twice as large as those based on F, and R- factors based on ALL data will be even larger.

### Fractional atomic coordinates and isotropic or equivalent isotropic displacement parameters (Å^2^)

**Table:**

	*x*	*y*	*z*	*U*_iso_*/*U*_eq_	
Br1	0.75214 (3)	0.53350 (5)	0.72206 (2)	0.07826 (14)	
Br2	0.67394 (2)	0.50575 (4)	0.31003 (2)	0.05547 (11)	
O	0.48779 (13)	0.7332 (2)	0.35440 (11)	0.0493 (4)	
H1O	0.4316	0.7816	0.3682	0.074*	
N	0.35471 (16)	0.8850 (2)	0.46645 (16)	0.0460 (5)	
C1	0.0119 (3)	0.7850 (6)	0.3456 (3)	0.0974 (13)	
H1	−0.0168	0.7738	0.2810	0.117*	
C2	−0.0382 (3)	0.7132 (5)	0.4196 (4)	0.0908 (12)	
H2	−0.1027	0.6544	0.4063	0.109*	
C3	0.0041 (3)	0.7256 (5)	0.5129 (3)	0.0836 (11)	
H3	−0.0311	0.6754	0.5638	0.100*	
C4	0.0993 (2)	0.8125 (4)	0.5333 (2)	0.0612 (7)	
H4	0.1286	0.8189	0.5979	0.073*	
C5	0.15180 (19)	0.8895 (3)	0.46034 (19)	0.0450 (6)	
C6	0.1066 (2)	0.8759 (5)	0.3656 (2)	0.0778 (10)	
H6	0.1398	0.9281	0.3144	0.093*	
C7	0.2567 (2)	0.9840 (3)	0.4827 (3)	0.0575 (7)	
H7A	0.2572	1.0781	0.4407	0.069*	
H7B	0.2591	1.0200	0.5508	0.069*	
C8	0.41771 (19)	0.8452 (3)	0.54029 (19)	0.0426 (6)	
H8	0.4014	0.8824	0.6025	0.051*	
C9	0.51473 (17)	0.7434 (3)	0.53116 (16)	0.0353 (5)	
C10	0.54482 (17)	0.6901 (3)	0.43760 (16)	0.0361 (5)	
C11	0.63563 (18)	0.5885 (3)	0.43328 (16)	0.0359 (5)	
C12	0.69736 (18)	0.5414 (3)	0.51723 (17)	0.0401 (5)	
H12	0.7580	0.4736	0.5127	0.048*	
C13	0.66716 (19)	0.5970 (3)	0.60777 (17)	0.0418 (5)	
C14	0.57710 (19)	0.6958 (3)	0.61547 (17)	0.0422 (5)	
H14	0.5577	0.7311	0.6772	0.051*	

### Atomic displacement parameters (Å^2^)

**Table:**

	*U*^11^	*U*^22^	*U*^33^	*U*^12^	*U*^13^	*U*^23^
Br1	0.0719 (2)	0.1044 (3)	0.05549 (19)	0.01255 (19)	−0.02322 (15)	0.00917 (17)
Br2	0.05471 (18)	0.0672 (2)	0.04574 (16)	0.00181 (13)	0.01445 (12)	−0.00697 (12)
O	0.0414 (9)	0.0656 (12)	0.0408 (9)	0.0077 (8)	0.0003 (7)	0.0096 (8)
N	0.0298 (10)	0.0410 (12)	0.0675 (14)	−0.0021 (8)	0.0053 (9)	0.0040 (10)
C1	0.053 (2)	0.156 (4)	0.082 (3)	0.000 (2)	−0.0136 (18)	−0.022 (3)
C2	0.0439 (18)	0.100 (3)	0.129 (4)	−0.0135 (19)	0.009 (2)	−0.029 (3)
C3	0.064 (2)	0.079 (3)	0.109 (3)	−0.0202 (18)	0.026 (2)	0.010 (2)
C4	0.0560 (17)	0.0622 (19)	0.0658 (18)	−0.0063 (14)	0.0082 (14)	0.0068 (14)
C5	0.0305 (12)	0.0431 (14)	0.0618 (15)	0.0078 (10)	0.0070 (11)	0.0023 (12)
C6	0.0477 (17)	0.121 (3)	0.065 (2)	0.0031 (18)	0.0077 (15)	0.0122 (19)
C7	0.0386 (13)	0.0434 (16)	0.091 (2)	0.0027 (11)	0.0084 (14)	−0.0010 (14)
C8	0.0348 (12)	0.0384 (13)	0.0555 (15)	−0.0075 (10)	0.0107 (11)	−0.0058 (11)
C9	0.0293 (11)	0.0349 (12)	0.0420 (12)	−0.0083 (9)	0.0040 (9)	−0.0002 (10)
C10	0.0310 (11)	0.0368 (12)	0.0406 (12)	−0.0077 (9)	0.0036 (9)	0.0040 (10)
C11	0.0321 (11)	0.0367 (13)	0.0394 (12)	−0.0079 (9)	0.0075 (9)	−0.0003 (10)
C12	0.0316 (12)	0.0364 (13)	0.0522 (14)	−0.0026 (9)	0.0016 (10)	0.0033 (10)
C13	0.0356 (12)	0.0479 (15)	0.0407 (12)	−0.0065 (11)	−0.0070 (9)	0.0050 (11)
C14	0.0391 (13)	0.0480 (14)	0.0394 (12)	−0.0102 (11)	0.0025 (10)	−0.0060 (10)

### Geometric parameters (Å, °)

**Table:**

Br1—C13	1.889 (2)	C5—C6	1.375 (4)
Br2—C11	1.890 (2)	C5—C7	1.509 (4)
O—C10	1.340 (3)	C6—H6	0.9300
O—H1O	0.8200	C7—H7A	0.9700
N—C8	1.269 (3)	C7—H7B	0.9700
N—C7	1.469 (3)	C8—C9	1.458 (3)
C1—C2	1.343 (5)	C8—H8	0.9300
C1—C6	1.386 (5)	C9—C14	1.393 (3)
C1—H1	0.9300	C9—C10	1.412 (3)
C2—C3	1.343 (5)	C10—C11	1.390 (3)
C2—H2	0.9300	C11—C12	1.384 (3)
C3—C4	1.375 (4)	C12—C13	1.381 (3)
C3—H3	0.9300	C12—H12	0.9300
C4—C5	1.364 (4)	C13—C14	1.373 (3)
C4—H4	0.9300	C14—H14	0.9300
			
C10—O—H1O	109.5	C5—C7—H7B	109.5
C8—N—C7	118.8 (2)	H7A—C7—H7B	108.1
C2—C1—C6	119.9 (3)	N—C8—C9	122.4 (2)
C2—C1—H1	120.1	N—C8—H8	118.8
C6—C1—H1	120.1	C9—C8—H8	118.8
C1—C2—C3	120.5 (3)	C14—C9—C10	119.7 (2)
C1—C2—H2	119.8	C14—C9—C8	119.8 (2)
C3—C2—H2	119.8	C10—C9—C8	120.5 (2)
C2—C3—C4	120.1 (3)	O—C10—C11	119.9 (2)
C2—C3—H3	119.9	O—C10—C9	121.9 (2)
C4—C3—H3	119.9	C11—C10—C9	118.2 (2)
C5—C4—C3	121.2 (3)	C12—C11—C10	121.9 (2)
C5—C4—H4	119.4	C12—C11—Br2	118.59 (17)
C3—C4—H4	119.4	C10—C11—Br2	119.44 (16)
C4—C5—C6	117.6 (3)	C13—C12—C11	118.8 (2)
C4—C5—C7	121.2 (3)	C13—C12—H12	120.6
C6—C5—C7	121.2 (3)	C11—C12—H12	120.6
C5—C6—C1	120.7 (3)	C14—C13—C12	121.2 (2)
C5—C6—H6	119.7	C14—C13—Br1	120.20 (18)
C1—C6—H6	119.7	C12—C13—Br1	118.63 (18)
N—C7—C5	110.7 (2)	C13—C14—C9	120.2 (2)
N—C7—H7A	109.5	C13—C14—H14	119.9
C5—C7—H7A	109.5	C9—C14—H14	119.9
N—C7—H7B	109.5		
			
C6—C1—C2—C3	−1.5 (7)	C8—C9—C10—O	1.5 (3)
C1—C2—C3—C4	0.1 (6)	C14—C9—C10—C11	1.1 (3)
C2—C3—C4—C5	1.0 (5)	C8—C9—C10—C11	−177.7 (2)
C3—C4—C5—C6	−0.6 (5)	O—C10—C11—C12	179.7 (2)
C3—C4—C5—C7	−179.6 (3)	C9—C10—C11—C12	−1.1 (3)
C4—C5—C6—C1	−0.9 (5)	O—C10—C11—Br2	−2.4 (3)
C7—C5—C6—C1	178.1 (3)	C9—C10—C11—Br2	176.78 (15)
C2—C1—C6—C5	2.0 (6)	C10—C11—C12—C13	0.3 (3)
C8—N—C7—C5	−112.6 (3)	Br2—C11—C12—C13	−177.68 (17)
C4—C5—C7—N	95.3 (3)	C11—C12—C13—C14	0.7 (3)
C6—C5—C7—N	−83.7 (3)	C11—C12—C13—Br1	−179.63 (17)
C7—N—C8—C9	178.1 (2)	C12—C13—C14—C9	−0.6 (4)
N—C8—C9—C14	−176.2 (2)	Br1—C13—C14—C9	179.65 (17)
N—C8—C9—C10	2.6 (3)	C10—C9—C14—C13	−0.3 (3)
C14—C9—C10—O	−179.7 (2)	C8—C9—C14—C13	178.5 (2)

### Hydrogen-bond geometry (Å, °)

**Table:**

*D*—H···*A*	*D*—H	H···*A*	*D*···*A*	*D*—H···*A*
C7—H7A···Cg^i^	0.97	2.88	3.526 (3)	125
O—H1O···N	0.82	1.88	2.601 (3)	147

Symmetry codes: (i) −*x*+1, −*y*+2, −*z*+1.
